# System biology of bacterial cellulose production

**DOI:** 10.1186/1753-6561-8-S4-P256

**Published:** 2014-10-01

**Authors:** Samara Silva de Souza, Luismar Marques Porto

**Affiliations:** 1 Department of Chemical and Food Engineering, Federal University of Santa Catarina, Florianópolis, Santa Catarina, Integrated Technologies Laboratory (InteLab), Genomic and Tissue Engineering Group, Florianópolis, SC, Brazil

## Background

Reconstruction and applications of genome scale metabolic models have truly influenced the field of systems biology [1,2]. These models have a promising ability to describe cellular phenotypes accurately and to relate the annotated genome sequence to the physiological functions of a cell [3,4]. *Gluconacetobacter *has been extensively characterized and is a model system for cellulose biosynthesis [5]. Using high throughput sequencing technologies a high quality draft assembly of the genome of this bacteria, *Gluconacetobacter hansenii *ATCC 23769 (GenBank number: CM000920 and taxonomy ID: 714995), has been completed and can be used to understand how this bacteria is able to produce cellulose. Bacterial cellulose has been used in the medical field as wound dressing and artificial skin material. Computational tools for predicting fluxes in biochemical networks are applied in the fields of integrated systems biology, bioinformatics, and genomics.

## Methods

The reconstruction process involves the following steps: (1) creation of a draft model; (2) manual curation; (3) conversion into a mathematical format; (4) identification and filling of gaps; and (5) simulation and visualization. The annotated data of the genome sequence of *G. hansenii *were used as the input for the Pathway Tools software, which can mapping genes to reactions in an automated manner. Moreover, a resource called Model SEED was used by creating a database of the organism. Then, to correct any mistakes and improve the reconstruction, the manual curation must be done using data from many different sources. An essential step is the addition of reactions which are not inferred from genome annotation and will decrease the number of dead-ends. The metabolic network file was loaded into MATLAB^® ^using functions available in the COBRA toolbox. Moreover, a set of computational systems biology tools, written by our group called GEnSys (Genomic Engineering System), was applied to the network. The GEnSys comprises several modules that allow analysis and simulation of biochemical reaction networks, such as FBA (flux balance analysis) and MFA (metabolic flux analysis).

## Results and conclusions

In this work, we present an initial draft of genome-scale metabolic reconstruction and network analysis of *G. hansenii*. Using the model in conjunction with constraint-based methods, we simulated the metabolic fluxes induced by three different environmental conditions to understand the effects of different carbons sources: glucose, glycerol and mannitol on cellulose production. We decided to maximize cellulose to evaluate the results. Also the maximum yield can be calculated for each carbon source. We summarized the biochemistry of each reaction in the *G. hansenii *model and we focused in the central pathways and cellulose biosynthesis to choose the reactions and metabolites in the core model. The core is a small scale model that can be used for testing and evaluating new constraint-based analysis methods, but still can be updated. This simplified model is representative and elevated our capability for understanding and predicting the cellular behavior of *G. hansenii *under any perturbations.
